# Impact of surgeon experience on corneal endothelial changes after cataract surgery

**DOI:** 10.5935/0004-2749.2024-0368

**Published:** 2025-06-24

**Authors:** Umberto Antonini Rizzuto, Larissa Lima Magalhães, Bruno Lovaglio Cançado Trindade

**Affiliations:** 1 Instituto de Olhos Ciências Médicas de Minas Gerais, Belo Horizonte, MG, Brazil; 2 Faculdade de Medicina Ciências Médicas de Minas Gerais, Belo Horizonte, MG, Brazil

**Keywords:** Cataract extraction, Phacoemulsification, Endothelium, corneal, Lens implantation, intraocular, Visual acuity, Internship and residency, Surgeons

## Abstract

**Purpose:**

To compare endothelial corneal cell changes following cataract surgery performed by phacoemulsification with intraocular lens implantation, conducted by surgeons with varying levels of experience.

**Methods:**

Two hundred and eighty-three eyes diagnosed with cataract were included. Lens opacity was classified into three categories (I, II, and III). Surgeons were categorized into four experience levels (1, 2, 3, and 4), based on years of practice and lifetime surgeries performed. Corneal endothelial characteristics were assessed using non-contact specular microscopy, with measurements taken before surgery and 30-60 days post-surgery.

**Results:**

Preand postoperative endothelial analysis showed no significant differences between surgeon levels regarding visual acuity achieved, corneal thickness, and endothelial hexagonality. However, the central endothelial cell density index showed a significantly greater reduction among level 1 surgeons (p=0.026). Grade II cataracts exhibited significant variations in the central endothelial cell density (p=0.011) and average cell size, with level 1 surgeons showing the largest increases (p=0.024).

**Conclusions:**

The analysis revealed significant differences in visual acuity and endothelial indices between surgeon experience levels, with less experienced surgeons showing greater variations and poorer performance. Clinical protocols should consider these data to establish safer training protocols.

## INTRODUCTION

The corneal endothelium, a monolayer of polygonal cells on the cornea’s posterior surface, plays a crucial role in maintaining corneal transparency by regulating the fluid and solute balance between the corneal stroma and the anterior chamber. The endothelium’s primary source of oxygen is the aqueous humor, and its key function is to prevent excess fluid accumulation in the stroma, thereby preserving corneal transparency^(1)^.

Aging leads to a gradual decline in the number of endothelial cells, resulting in a decrease in cell density and alterations in cell morphology. These changes can be monitored using specular microscopy^(2)^. Furthermore, certain conditions, such as cataract surgery-especially phacoemulsification-can exacerbate endothelial cell loss, compromising endothelial function and corneal clarity. The extent of cell loss is also influenced by factors like excessive ultrasound energy during surgery and the surgeon’s level of experience.

According to Santhiago et al.^(3)^, a thorough analysis of the corneal endothelium is crucial for the success of cataract surgery and maintaining corneal transparency. Notably, bullous keratopathy (BK) has been linked to phacoemulsification.

Specular microscopy is an important technique for evaluating endothelial health, providing information on cell density, polymegathism (variation in cell size), and pleomorphism (variation in cell shape). This technique is also helpful in screening donor tissue for corneal transplantation and monitoring patients at risk of developing BK, a condition characterized by significant endothelial cell loss, leading to fluid buildup, edema, and reduced corneal transparency^(4^-^6)^.

Bullous keratopathy is a significant complication that can occur after cataract surgery or other intraocular procedures, and it is one of the leading indications for corneal transplantation^(7^,^8)^. The risk of developing BK increases with age, particularly in patients over 60 years old. In addition to surgical factors, endothelial dystrophies, such as Fuchs’ dystrophy, can also lead to endothelial dysfunction^(9)^.

Evaluating corneal endothelial health is vital for monitoring corneal transparency and preventing postoperative complications, with specular microscopy serving as a valuable tool for this monitoring.

The acceleration of endothelial loss is directly related to various surgical factors, such as the amount of ultrasound energy used for nuclear cataract fragmentation, the proximity of the phacoemulsification handpiece to the corneal endothelium, and the use of adjuvant substances such as adrenaline for pupil dilation and trypan blue for anterior capsule staining. Inadequate viscoelastics, intraocular manipulation, and fluid dynamics for maintaining the anterior chamber also contribute to endothelial loss. Furthermore, the experience level of the surgeon may also impact the degree of endothelial damage^(10^-^12)^.

The use of trypan blue and adrenaline is based on the specific requirements of each surgery and the individual preferences of each surgeon, with a possible trend toward more frequent use by less experienced surgeons.

Chamorro et al.^(11)^ identified several factors contributing to endothelial damage following phacoemulsification. However, the impact of the surgeon’s experience has been relatively underexamined.

This study investigated variations in endothelial indices following phacoemulsification based on the surgeon’s level of experience in an ophthalmology teaching setting. It also examined the relationship between surgeon experience and endothelial cell loss during phacoemulsification with intraocular lens implantation. Our findings may help inform the development of a protocol for assigning surgeries to residents, fellows, and preceptors with varying experience levels.

## METHODS

This non-randomized, self-paired prospective study included 283 eyes with clinically significant cataracts examined between 1^st^ January 2024 and 10^th^ October 2024. All patients underwent comprehensive preoperative ophthalmic evaluations, including visual acuity measurements, tonometry, retinal indirect ophthalmoscopy, optical biometry (Zeiss IOL Master 500), and specular microscopy. Lens density was assessed using slit-lamp examination and classified according to the Lens Opacities Classification System III (LOCS III), which includes nuclear opalescence (NO), nuclear color, posterior subcapsular cataract (P), and cortical cataract (P). Eyes were classified into three groups based on increasing lens density using the LOCSA adaptation. The groups were defined as follows: Group I included mild opacifications and densities (NO1-NO2; NC1-NC2), Group II included moderate opacifications and densities (NO3-NO4; NC3-NC4; P5, C5), and Group III included advanced opacifications and densities (NO5-NO6; NC5-NC6).

Endothelial analysis was conducted using a non-contact specular microscope (Tomey EM-4000) to assess cell density, average cell size, percentage of hexagonal cells, and central corneal thickness. Measurements were obtained preoperatively and between 30 and 60 days postoperatively.

Surgeries were performed by surgeons categorized into 4 groups based on experience: level 1 (10-50 surgeries), level 2 (60-200 surgeries), level 3 (210-500 surgeries), and level 4 (over 1000 surgeries).

Phacoemulsification with intraocular lens implantation was performed under peribulbar block, utilizing trypan blue, hydroxypropyl methylcellulose, adrenaline, and Ringer’s lactate solution. Postoperative evaluations occurred on days 1, 7, 30, and optionally on day 60, with a comprehensive follow-up assessment at the final visit.

### Inclusion criteria

Patients who qualified the following criteria were eligible for inclusion: 1) Clinically significant cataract of any etiology; 2) corrected visual acuity of 0.67 or worse; 3) age between 40 and 100 years; 4) ability to undergo preand postoperative examinations; 5) provision of written informed consent.

The exclusion criteria were as follows: 1) presence of other concomitant ocular conditions that could affect endothelial analysis (such as glaucoma, uveitis, or endothelial dystrophies); 2) chronic use of topical medications, except preservative-free ocular lubricants deemed safe for the corneal endothelium; 3) chronic contact lens wear; 4) endothelial cell counts below 1,000 cells/mm^2^ on specular microscopy.

Data collection was performed by one of the authors (LLM).

This study was approved by the institutional ethics committee and adhered to the principles of the Declaration of Helsinki. All patients provided written informed consent.

### Statistical analysis

Due to non-normal distribution and variance homogeneity, assessed by the Kolmogorov-Smirnov test, nonparametric tests were employed. The Mann-Whitney and Kruskal-Wallis tests were used to compare independent groups, while the Wilcoxon test was used to compare paired observations (preand postoperative). Categorical variables were analyzed using Pearson’s Chi-square test, with Monte Carlo simulation applied for categories with expected frequencies below five. A significance level of 5% was set, with p-values ≤0.05 considered significant. Statistical analyses were performed using SPSS version 25.0.

## RESULTS

The characteristics of the study population are summarized in table 1.

**Table 1:** Characteristics of the study population

Variables	n (%)
**Age**	
Median (P25-P75)	71 (66-76.25)
**Surgeon level**	
1	60 (21.2)
2	78 (27.6)
3	90 (31.8)
4	55 (19.4)
**LOCS A**	
I	10 (3.5)
II	205 (72.4)
III	68 (24)

There was no significant difference in the age distribution across different surgeon levels (p=0.143, Table 2). However, a statistically significant difference was observed in the distribution of LOCSA classification (p=0.007). Surgeons at levels 3 and 4 operated on a higher proportion of eyes in the more advanced categories (Category III), while surgeons at levels 1 and 2 performed a higher proportion of surgeries in Category II.

**Table 2:** Distribution of age and LOCSA classification according to surgeon experience level

Variables	Surgeon level				p-value
	1 (n=60)	2 (n=78)	3 (n=90)	4 (n=55)	
**Age**					
Median (IQR)	70 (66-74)	70 (65-73)	71 (66-77)	73 (66-79)	0.143^*^
**LOCSA**					
I	2 (3.3%)	4 (5.1%)	3 (3.3%)	1 (1.8%)	0.07^**^
II	47 (78.3%)	66 (84.6%)	58 (64.4%)	34 (61.8%)	
III	11 (18.3%)	8 (10.3%)	29 (32.2%)	20 (36.4%)	

^*^= Kruskal-Wallis; ^**^= Qui-quadrado via Monte Carlo.

IQR= interquartile range.

Table 3 presents a detailed comparison of visual acuity and endothelial indices of patients, both preoperatively and between 30 and 60 days postoperatively, according to surgeon level.

**Table 3:** Comparison of visual acuity and endothelial indices according to surgeon experience level

Variables	Surgeon level				p-value^*^
	1	2	3	4	
**Preoperative measures**	n=60	n=78	n=90	n=55	
**AVPre**					
Median (IQR)	0.30 (0.12-0.50)	0.40 (0.30-0.50)	0.30 (0.13-0.50)	0.30 (0.06-0.50)	**0.040**
**CDPre**					
Median (IQR)	2516 (2382-2696)	2521 (2331-2700)	2447 (2309-2612)	2431 (2229-2604)	**0.044**
**AVGPre**					
Median (IQR)	395 (370-421)	398 (371-429)	404 (383-433)	411 (384-449)	0.076
**6APre**					
Median (IQR)	44 (40-49)	45 (39-49)	43 (39-48)	42 (37-47)	0.158
**CCTPre**					
Median (IQR)	522 (494-550)	514 (491-548)	536 (505-556)	528 (503-564)	0.120
**Postoperative measures**	n=57	n=60	n=60	n=60	
**AVPOS**					
Median (IQR)	1.00 (0.90-1.00)	1.00 (0.67-1.00)	1.00 (0.67-1.00)	0.90 (0.67-1.00)	0.215
**CD30d**					
Median (IQR)	1987 (1432-2283)	2157 (1557-2355)	2068 (1505-2349)	1644 (1402-2192)	**0.025**
**AVG30d**					
Median (IQR)	503 (437-699)	462 (421-642)	480 (426-656)	555 (450-713)	0.061
**6A30d**					
Median (IQR)	38 (35-45)	39 (35-44)	39 (35-44)	37 (33-45)	0.670
**CCT30d**					
Median (IQR)	530 (499-557)	520 (484-547)	530 (505-554)	533 (496-568)	0.246

* Kruskal-Wallis test

Multiple comparisons adjusted by Bonferroni:

AV pre: surgeon level 2 vs 3: p=0.040, other levels: p>0.05.

CD pre: surgeon level 1 vs 4: p=0.019 and 2 vs 4: p=0.033; other levels: p>0.05

CD 30d: surgeon level 2 vs 4: p=0.028; other levels: p>0.05.

AVpre= preoperative visual acuity; CDPre= preoperative central endothelial density; CCTPre= preoperative central corneal thickness; AVPOS= postoperative visual acuity; CD30d= central endothelial density between 30 and 60 days after surgery.

Median preoperative visual acuity (AVPre) ranged from 0.30 to 0.40, with a significant difference in distribution among surgeon levels (p=0.040). Multiple comparison analysis revealed a statistically significant difference between levels 2 and 3 (p=0.040), while other comparisons showed no significant difference.

Preoperative central endothelial density (CDPre) also varied significantly among surgeon levels (p=0.044). Specifically, significant differences were observed between levels 1 and 4 (p=0.019) and between levels 2 and 4 (p=0.033), with no significant differences in other comparisons for this variable.

Preoperative central corneal thickness (CCTPre) and other indices (AVGPre and 6APre) did not differ significantly among surgeon levels, with p-values above 0.05 (p=0.076, p=0.158, and p=0.120, respectively).

Postoperative visual acuity (AVPOS) reached a median of 1.00 across nearly all surgeon levels, with no significant difference between groups (p=0.215). However, central endothelial density between 30 and 60 days after surgery (CD30d) differed significantly among surgeon levels (p=0.025). Multiple comparison analysis revealed a significant difference between surgeon levels 2 and 4 (p=0.028), while other comparisons were not significant.

The variables AVG30d, 6A30d, and CCT30d showed no significant differences between surgeon levels (p=0.061, p=0.670, and p=0.246, respectively).

Table 4 compares the variation in visual acuity (DeltaAV) and endothelial indices (DeltaCD, DeltaAVG, Delta6A, and DeltaCCT) between eyes operated on by surgeons of different levels in LOCSA grade II and III, excluding LOCSA grade I.

**Table 4:** Comparison of the variation in visual acuity and endothelial indices between different levels of surgeon experience in LOCSA grade II and LOCSA grade III groups

Variables	LOCSA II				p-value^*^
	Surgeon level				
	1 (n=45)	2 (n=64)	3 (n=53)	4 (n=30)	
**DeltaAV**					
Median (IQR)	0.50 (0.40-0.60)	0.50 (0.37-0.70)	0.55 (0.50-070)	0.50 (0.30-0.60)	0.363
**DeltaCD**					
Median (IQR)	-548 (-1095, -270)	-307.5 (-728, -119)	-245 (-619, -104)	-513.50 (-1028, -183)	**0.011**
**DeltaAVG**					
Median (IQR)	105 (42-315)	49.5 (25-158)	43 (17-160)	79 (22-276)	**0.024**
**Delta6A**					
Median (IQR)	44 (39-49)	45 (40-49)	43 (39-48)	42 (37-48)	0.331
**DeltaCCT**					
Median (IQR)	6 (-6-17)	-0.5 (-8-7)	0.0 (-10-7)	1.5 (-5-10)	0.090
**Variables**	**LOCSA III**				p-value^*^
	**Surgeon level**				
	**1 (n=10)**	**2 (n=8)**	**3 (n=26)**	**4 (n=20)**	
**DeltaAV**					
Median (IQR)	0.75 (0.60-0.80)	0.55 (0.35-0.68)	0.61 (0.47-0.87)	0.70 (0.55-0.89)	0.315
**DeltaCD**					
Median (IQR)	-839 (-1048, -397)	-863 (-923, -599.5)	-596 (-1012, -189)	-613 (-946.5, -78)	0.640
**DeltaAVG**					
Median (IQR)	251 (106, 361)	236 (102, 307.5)	142 (52, 345)	131 (28.5, 305)	0.777
**Delta6A**					
Median (IQR)	42 (40-49)	38 (33-43)	44 (40-47)	42.5 (36.5-44)	0.165
**DeltaCCT**					
Median (IQR)	-2 (-8-12)	1.5 (-3-7.5)	1 (-5-9)	1.5 (-9.5-9)	0.978

* Kruskal-Wallis test

Adjusted Bonferroni multiple comparisons:

DeltaCD: Surgeon level 1 vs 2: p=0.013, 1 vs 3: p=0.002, others: p>0.05

DeltaAVG: Surgeon level 1 vs 2: p=0.018, 1 vs 3: p=0.004, others: p>0.05

DeltaAV= variation in visual acuity; DeltaCD= variation in central endothelial density; DeltaCCT= variation in central corneal thickness.

### LOCSA grade II:

- DeltaAV: The median variation in visual acuity ranged from 0.50 to 0.55, with no significant difference between surgeon levels. There was no significant difference between surgeon levels (p=0.363), indicating no significant impact of surgeon’s experience on visual acuity in the LOCSA grade II group.
- DeltaCD: The variation in central endothelial density showed a significant difference between surgeon levels (p=0.011), with lower medians in eyes operated by surgeons at levels 1 and 4 compared to others. Adjusted multiple comparisons revealed significant differences between surgeons levels 1 and 2 (p=0.013) and 1 and 3 (p=0.002), suggesting a greater impact of level 1 surgeons on endothelial density.
- DeltaAVG: A significant difference was also observed in DeltaAVG (p=0.024), with higher variation in eyes operated by level 1 surgeons compared to levels 2 and 3. Multiple comparisons revealed significant differences between levels 1 and 2 (p=0.018) and levels 1 and 3 (p=0.004).
- Delta6A and DeltaCCT: No significant differences were observed between surgeon levels for Delta6A (p=0.331) and DeltaCCT (p=0.090), suggesting no substantial impact of surgeon’s experience on these endothelial and structural parameters.

### LOCSA grade III

- DeltaAV: A trend of higher median variations in visual acuity was observed between eyes operated by different levels of surgeons, particularly for level 1 (median 0.75) and level 4 (median 0.70), although it was not statistically significant (p=0.315).
- DeltaCD: Similar to the LOCSA II group, DeltaCD showed high negative values across surgeon levels, with no significant difference between levels (p=0.640).
- DeltaAVG: Less experienced surgeons (levels 1 and 2) had higher medians for DeltaAVG in the eyes operated, but the between-group differences were not significant (p=0.777).
- Delta6A and DeltaCCT: Consistent with the LOCSA grade II group, no significant differences were found in Delta6A and DeltaCCT between eyes operated by different levels of surgeons (p=0.165 and p=0.978, respectively).

## DISCUSSION

Cataract surgery with intraocular lens implantation is a safe and effective procedure, but it carries risks, including endothelial corneal injury. This complication can lead to corneal decompensation, posing challenges for patients, physicians, and healthcare services^(7)^.

A comparative analysis of visual acuity and endothelial indices in patients, both preoperatively and postoperatively, according to surgeon experience levels, revealed a significant difference in the distribution of preoperative median visual acuity between surgeon experience levels, consistent with previous studies^(10^,^12^,^13)^. Additionally, preoperative central endothelial density showed significant variation in distribution between surgeon groups, indicating a potential bias in the referral of eyes with more compromised corneas to more experienced surgeons. This may have led to worse outcomes for this group of surgeons. Other preoperative corneal indices showed no significant differences between surgeon experience levels.

Postoperative results revealed a median visual acuity of 1.00 across all surgeon experience levels, indicating substantial improvement and no inter-group differences. However, postoperative central endothelial density differed significantly between surgeon levels, with more experienced surgeons having lower endothelial density results. The variation in central endothelial density showed significant differences between surgeon levels, with level 1 surgeons performing worse compared to levels 2 and 3. Other variables did not exhibit significant differences between surgeon levels, indicating that these endothelial and structural measures were largely unaffected by surgeon experience, with the exception of endothelial density.

In the LOCSA grade II group, significant differences were observed in two parameters. First, the variation in central endothelial density was lower in eyes operated on by surgeons at levels 1 and 4 compared to other levels, with significant differences between levels 1 and 2, and levels 1 and 3, suggesting a greater impact of level 1 surgeons’ experience on endothelial density. Second, the variation in average cell size was higher in eyes operated by level 1 surgeons compared to levels 2 and 3.

Although most surgical complications, including the most common complication of posterior capsule rupture, do not typically vary significantly by surgeon experience level in teaching institutions, according to Barreto Junior, Jacson et al.^(13)^, our findings suggest that surgeon experience can have a significant impact on postoperative outcomes, especially in eyes with LOCSA grade II cataracts.

Greater losses in endothelial density indicate more extensive intraoperative endothelial injury, which is also associated with an increase in average cell size, as remaining cells tend to enlarge to occupy the space left by lost adjacent cells.

Analyzing the performance differences between surgeon groups based on changes in corneal parameters before and after surgery, we found significantly greater deterioration in endothelial density and average cell size in group 1 compared to groups 2 and 3, indicating that less experienced surgeons showed the greatest variations. This finding raises questions about the underlying reasons, which may include greater manipulation of intraocular instruments, longer surgery times, more frequent use of adjunctive substances like dyes and mydriatics, and greater use of ultrasonic energy. Therefore, it would be prudent to consider not only variables like lens density, patient status, associated ocular pathologies, and previous visual acuity, but also to direct surgeries for eyes with more compromised endothelial and cellular characteristics to more experienced surgeons, incorporating these findings into the cataract surgery distribution protocols in teaching institutions.

Analysis of visual outcomes and corneal endothelial characteristics following cataract surgery performed by surgeons with varying levels of experience showed significant improvements in visual acuity and an increa-se in the average endothelial cell size. However, the procedure also resulted in a reduction in hexagonality and endothelial cell density, as well as an increase in the average cell size. While pachymetry showed a slight increase, this change was not statistically significant.

Further research is necessary to better understand the differences in corneal changes attributed to surgeons with varying experience levels. A randomized controlled trial would likely provide a better understanding of the relationship between surgeon experience and corneal outcomes.

From an educational perspective, a key focus of our institution, we emphasize the importance of instructors mastering not only manual skills but also techniques and technology to be effective. Mastery of techniques and technology enables surgeons to improve more efficiently, beyond just manual proficiency. Investing in the qualification of mentors or instructors is essential, and teaching should be structured, combining theoretical classroom instruction with supervised practical experience. Furthermore, evidence-based medicine should serve as the foundation for learning, and instructors should be able to critically analyze the scientific information they convey^(14)^.
